# Community views about routine HIV testing and antiretroviral treatment in Botswana: signs of progress from a cross sectional study

**DOI:** 10.1186/1472-698X-7-5

**Published:** 2007-06-08

**Authors:** Anne Cockcroft, Neil Andersson, Deborah Milne, Thamie Mokoena, Mokgweetsi Masisi

**Affiliations:** 1 CIET Trust, 71 Oxford Road, Saxonwold, Johannesburg 2196, South Africa; 2 Centro de Investigación de Enfermedades Tropicales (CIET), Universidad Autónoma de Guerrero, Apdo 2-25, Acapulco, Mexico; 3 CIET Trust, PO Box 1240, Gaborone, Botswana

## Abstract

**Background:**

The Botswana government began providing free antiretroviral therapy (ART) in 2002 and in 2004 introduced routine HIV testing (RHT) in government health facilities, aiming to increase HIV testing and uptake of ART. There have been concerns that the RHT programme might be coercive, lead to increased partner violence, and drive people away from government health services.

**Methods:**

We conducted a household survey of 1536 people in a stratified random sample of communities across Botswana, asking about use and experience of government health services, views about RHT, views about ART, and testing for HIV in the last 12 months. Focus groups further discussed issues about ART.

**Results:**

Some 81% of respondents had visited a government clinic within the last 24 months. Of these 92% were satisfied with the service, 96% felt they were treated with respect and 90% were comfortable about confidentiality. Almost all respondents said they would choose a government clinic for treatment of AIDS.

Nearly one half (47%) thought they were at risk of HIV. Those who had experienced partner violence within the last 12 months were more likely to think themselves at risk. One half of those who had visited a government facility in the last 24 months were offered HIV tests, and nearly half were tested. A few (8%) of those who were not asked thought they were tested. Most people (79%) had heard of RHT and 94% were in favour of it. Over one half (55%) of the entire sample had been tested for HIV within the last 12 months, one half of these through RHT. Women were more likely to have been tested.

Nearly everyone (94%) had heard of ART and thought it could help AIDS. Focus groups identified problems of access to ART due to distance from treatment centres and long queues in the centres.

**Conclusion:**

Public awareness and approval of RHT was very high. The high rate of RHT has contributed to the overall high rate of HIV testing. The government's programme to increase HIV testing and uptake of ART is apparently working well. However, turning the tide of the epidemic will also require further concerted efforts to reduce the rate of new HIV infections.

## Background

The prevalence of HIV infection in Botswana remains amongst the highest in the world. Even after recent downward adjustments to figures from Botswana and other countries, an estimated 24.1% of the Botswana adult population (15–49 years) was HIV positive in 2005 [1]. In early 2002 the government of Botswana began providing free antiretroviral therapy (ART) for all patients with a CD4 count less than 200 or an AIDS-defining illness [2]. Enrolment into the programme was initially slow, with problems of shortage of staff, and people not coming forward for testing because of stigma and denial [3]. In order to increase the rate of HIV testing and subsequent enrolment into ART, in early 2004 the government introduced routine HIV testing (RHT) in government health facilities [4]. There is interest in the Botswana programme as a possible model for others to follow, but concerns have been raised about the implementation of routine testing, especially about the adequacy of counselling and informed consent [5]. A study in Botswana in 2005 indicated that most people were in favour of the routine testing policy when it was explained to them, even though many of them had not heard about the policy and few had been tested under the new scheme [6]. However, the same study also found that nearly half of respondents thought routine testing would mean people avoided going for medical care because of fear of testing, and two thirds of those who had been tested, through voluntary counselling and testing (VCT) or the routine scheme, thought they could not refuse the test [6].

In mid 2006, the policy of routine testing had been in place for more than two years. How has this worked and has it led to the hoped-for increased HIV testing and entry into the programme of ART? We interviewed 1536 people in households across Botswana to document their knowledge and views about routine HIV testing and ART, frequency and place of recent HIV testing, experience of visits to government health facilities, and views about the role of government health services in testing and treatment for HIV and AIDS. We also conducted focus group discussions to explore in more depth perceptions and experiences of ART.

## Methods

The overall project of which this study is a part – a multi-method staged enquiry into ART in Botswana, Lesotho and Swaziland – received approval from the CIET Trust ethical review committee in July 2005.

**Figure 1:**
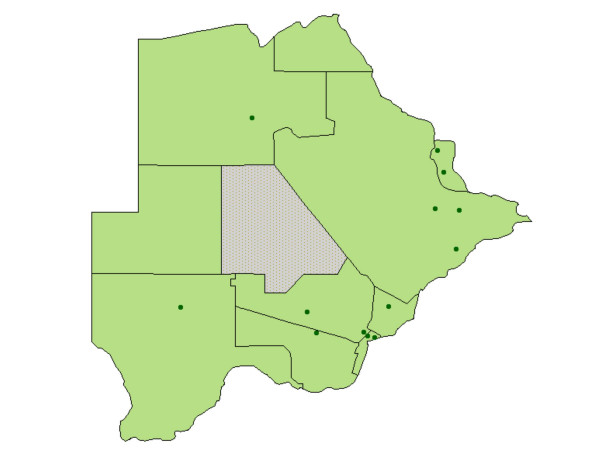
**Map of Botswana showing the location of the sample sites**. The dots show the sample sites. Most of the population of Botswana is concentrated in the south and east of the country. The grey area in the centre is a national game park in the Kalahari desert.

The 13 sites in the study were a random cluster sample, stratified by district and by rural/urban/capital location, of enumeration areas from the 2001 census. Within each enumeration area the site comprised 100 contiguous households, radiating out from a randomly selected starting point, with no sub-sampling within the site. This sampling strategy allows for multi-level analysis, relating community level variables to household and individual variables [7-9]. Figure 1 shows the location of the sample sites.

We developed a questionnaire for household respondents, drawing on our previous studies of knowledge and attitudes about HIV, HIV testing and ART in Southern Africa [10,11]. Local team members translated the English questionnaire into Setswana, with back-translation to check for any loss of meaning. The questionnaire format allowed data entry by electronic scanning. We trained local interviewers to administer the questionnaire to people over 18 years of age in each household, asking about use and perceptions of government and other health care providers, experience of RHT in government facilities, knowledge and views about the government policy of RHT in health facilities, HIV testing in the last 12 months, intention to have a test for HIV, and knowledge and views about ART. The questions about awareness of and views about RHT were in a separate section from the questions about actual experiences in government facilities. The interviewers briefly explained routine testing only to those few respondents who had not heard about it. The wording of the explanation was: "Routine HIV testing means that when somebody attends a government health facility with some kind of illness or for a routine check-up they are offered an HIV test. They have to give their consent to have the test and have the option of refusing. If they test positive they are offered counselling and appropriate treatment as necessary." The first person interviewed in each household also answered general questions about the household, mostly to identify the household socio-economic status.

Before administering the questionnaire, the interviewers explained to respondents the purpose of the study, explained that they did not have to answer any questions they did not want to and that they could stop the interview at any time, and sought their consent to proceed.

The field teams conducted the household interviews in August 2006. In each community, before beginning work, they sought the consent of the chief to undertake the survey. Usually a team could complete the interviews in the target of 100 households in each site in one day. We started interviews early in the mornings and returned in the late afternoons in order to include employed people in the sample. Trained local researchers coded the responses to open-ended questions. We used Remark software [12] to scan the questionnaire responses into a computer database.

At the time of the household interviews, the interviewers asked respondents if they would be interested to join a group to discuss the findings when the team came back to the community, and recorded contact details of people who were interested (separately from their responses to the household questionnaire). After preliminary analysis of basic frequencies of questionnaire responses, we designed a feedback focus group guide to present some of the important findings and to discuss them. The topic areas included ARVs (in particular difficulties with getting ARVs and any other difficulties for people taking ARVs), and choice of health care providers. Trained members of the original field teams returned to the 13 original communities in late August 2006 and facilitated and recorded three feedback focus groups in each one: one of adult males, one of adult females, and one of male and female youth. Each focus group had 8–12 participants, drawn from among the household respondents. The reporters took notes during the group discussions and prepared reports, together with the facilitators, including specific quotes where relevant. A small group from the research team read through the reports and defined emerging themes.

We categorised households as more vulnerable if there was no male member aged 18–60 years (about a quarter of the households). Other indicators of household socio-economic status were the type of roof, the occupation of the main breadwinner, whether there was enough food in the house in the last week, and whether the respondent considered the household income sufficient for their needs. We included household socioeconomic status in the analysis, since it may be related to HIV risk and is often related to access to and experience of health and other services [9,13].

We compared the proportions that lived in rural communities, urban communities, and the capital (Gaborone) in the sample population and the census population and calculated site weights to allow for differences in distribution between the sample and actual population. Table 1 shows how the weights were calculated. We used these weights to adjust the reported frequencies of variables at national level. We undertook this weighting because of the likelihood that there may be differences between urban and rural populations in the outcomes we were measuring.

**Table 1:** Calculation of site weights

Stratum	Census population	Proportion of census population (a)	Sample population	Proportion of sample population (b)	Site weight (a/b)
Rural	757,329	0.4569	731	0.4759	0.9601
Urban	718,480	0.4335	705	0.4590	0.9445
Capital	181,627	0.1096	100	0.0651	1.6832
Total	1,657,436		1536		

The census figures are for 2001. The sample population is the number of individuals interviewed in the households. The site weight for each group of sites is the proportion of the census population in those sites (a) divided by the proportion of the sample population in those sites (b). The proportions in rural and urban sites in the sample are close to those in the census population, while the proportion in the capital (Gaborone) in the sample is slightly lower than in the census population.

We used CIETmap [14] software to analyse the data. We examined the relationship between individual variables and the outcomes of considering oneself to be at risk of HIV and of having had an HIV test in the last 12 months. We used stratification to look for confounding and then performed logistic regression analysis to examine the combined effects of variables on the outcomes. We examined in univariate analysis the effects of those variables we considered, on the basis of previous work by ourselves and others, likely to be related to the outcomes of interest. For the logistic regression analysis of these variables we undertook a step-down from an initial model including all the variables to produce the final model.

## Results

### The household population

The field teams approached 2191 households. Of these, 632 (29%) had no one at home, 187 (9%) had no one over 18 years present, and 87 (4%) declined the interview. The final sample included 1285 households and in these we interviewed 1536 adults.

Table 2 shows household characteristics and Table 3 gives characteristics of the male and female interviewees. The proportions of men and women in paid employment in our sample were somewhat lower than the proportions in the overall population (over 20 years old), derived from 2001 census figures: 59% among men and 37% among women [15].

**Table 2:** Household characteristics

**Characteristic**	**Level**	**Weighted %**	**Unweighted %**	**Fraction**
Roof type	Tiles, corrugated iron	81.6	80.7	1023/1268
	Thatch, or shack	18.5	19.3	245/1268
Household size	Mean number of people	4.89 people	4.93 people	1282
Male presence	Male member 18–60 yrs	73.9	73.7	943/1279
Occupation of breadwinner	Unemployed	15.0	15.2	193/1268
	Pensioner, student	5.6	5.7	72/1268
	In some work	79.4	79.1	1003/1268
Enough food in last week	Yes	64.3	63.5	806/1270
Household income enough	Yes	31.1	30.3	386/1276

The denominator numbers indicate the number of households from which information was given for each characteristic, out of the total of 1285 households

**Table 3:** Characteristics of the household respondents

		**Total sample**		**Males**		**Females**	
**Characteristic**	**Level**	**Weighted %**	**Fraction**	**Weighted %**	**Fraction**	**Weighted %**	**Fraction**

Sex	Female	65.4	993/1512				
Age	Mean age	34.65 yr	1527	35.13 yr	517	34.63 yr	986
Marital status	Single	57.4	875/1533	57.6	292/518	58.0	575/993
	Cohabiting	18.4	290/1533	19.2	103/518	17.7	179/993
	Married	16.5	252/1533	17.8	94/518	15.2	150/993
	Separated, divorced, widowed	7.7	116/1533	5.3	29/518	9.15	87/993
Education	None and up to primary	42.2	661/1529	39.4	210/517	43.5	438/988
	Junior secondary and above	57.8	868/1529	60.6	307/517	56.5	550/988
Employment	Unemployed	52.9	815/1526	40.6	213/518	60.0	590/984
	Pensioner	2.3	36/1526	2.6	14/518	2.2	22/984
	Student	6.0	93/1526	7.3	38/518	5.2	52/984
	Some type of work	38.8	582/1526	49.6	253/518	33.0	320/984

The denominator numbers indicate the number of respondents who gave information for each characteristic, out of the total respondents and for males and females separately. Of the total 1536 respondents, sex was recorded for 1512: 993 women and 519 men.

### Use and experience of government health services

Respondents reported frequent use of government health services. One half (50.4%, 772/1534) said they had visited a government health facility for their own health care within the last three months. Three quarters had visited a government facility within the last 12 months (75.5%; 1157/1534) and 80.6% (1235/1534) had visited within the last 24 months (that is, since the policy of RHT was introduced into government clinics). The reported use of private health services was much lower: 5% of respondents had visited a private service in the last three months, 10% in the last 12 months, and 12% in the last 24 months. Men were significantly less likely than women to report a visit to a government health facility within the last 24 months (389/519 men visited compared with 828/991 women; OR 0.59, 95% CI 0.45–0.76).

Most people who reported using a government health facility said, in response to an open-ended question, that they chose a government facility because it was free or cheap (56.3%, 725/1294). Others mentioned the clinic was convenient or nearby (22.1%, 288/1294), or that the service was good (16.0%, 209/1294). Among those who reported a visit to a private health service within the last 24 months, most (68.9%, 122/176) said they chose a private service because they would get a better service or specialist treatment; no one said they chose the service because they wished to avoid HIV testing in a government clinic.

Nearly all those who had visited a government health facility were satisfied or very satisfied with the visit (91.6%; 1130/1233 for visits within 24 months; 91.8%; 707/771 for visits within three months). Similarly, nearly all those who had used a government health facility reported they had been treated with respect (95.5%; 1176/1231 for visits within 24 months; 95.8%; 738/771 for visits within three months). Most users of government health facilities said they were comfortable that the information the facility and staff had about them was kept private and confidential (89.5%; 1094/1222 for visits within 24 months; 89.7%; 683/764 for visits within three months).

In response to closed-ended questions, almost all respondents said they would advise someone to consult a government health centre for AIDS treatment (97.4%; 1486/1522) and almost all said they would go there themselves for AIDS treatment (97.8%; 1498/1528). There was no difference between men and women in the high proportion saying they would go to a government centre for AIDS treatment. In response to an open-ended question with more than one answer allowed, among those who said they would consult a government health centre for AIDS treatment, more than half said it was because they could get free or cheap treatment there (55.6%; 831/1494), over a quarter specifically mentioned good treatment and availability of ART (29.9%; 446/1494), and a further 12.6% (188/1494) mentioned generally good service.

Asked where they would go first if they had an illness they believed could be due to AIDS, the great majority said they would go to a government clinic or hospital (87.1%, 1341/1535), and a further 8.8% (134/1535) said they would go to a centre for voluntary counselling and testing for HIV (VCT). Of those who said they would go to a government clinic, 55% gave as a reason the availability of free medicines, including ART, and 50% mentioned good service, including HIV testing (more than one response was allowed to this open question).

### Perception of HIV risk and HIV testing

Nearly one half of the respondents thought they were at risk of HIV (47%; 725/1519). A number of factors were related to this perception in univariate analysis. The final model from logistic regression analysis is shown in Table 4. Some 10.5% (160/1527) of respondents had experienced violence from their partners during arguments in the last 12 months (women 11.2%, 110/986; men 8.6%, 45/517). People who had experienced partner violence were more than twice as likely to think themselves at HIV risk compared with people who had not experienced partner violence. People living in rural communities were also more likely to think themselves at risk of HIV, while people from households reporting their income was sufficient for their needs (31.1%, 386/1276) were *less *likely to think themselves at risk. Young people aged 18–24 years were only half as likely to think themselves at risk of HIV compared with people aged 25 years and older. Women were slightly more likely than men to think themselves at risk of HIV infection but the univariate association was not significant at the 5% level (OR 0.81, 95% CI 0.66–1.01), and the confidence interval became wider in the multivariate analysis.

**Table 4:** Logistic regression analysis of variables related to respondents believing themselves at risk of getting HIV

Variables	Crude OR	Adjusted OR	95% CI of adjusted OR	Mantel-Haenszel chi-square
Rural location	1.56	1.31	1.05–1.63	5.93
Household income sufficient for needs	0.43	0.46	0.36–0.58	40.78
Less than 25 years old	0.52	0.53	0.41–0.67	27.79
Beaten by partner in last 12 months	2.63	2.69	1.88–3.85	29.51

The variables included in the original saturated model were: rural/urban location, type of roof construction, household food sufficiency, income sufficiency, sex of respondent, age group, marital status, education level, and whether beaten by partner.

We asked respondents who had visited a government health facility within the last 24 months (since August 2004) about their experience of HIV testing when they visited the facility (Table 5). Nearly half (46.6%) of those who had visited a government health facility since August 2004 reported they were tested. Most (83.5%) of those asked about testing (offered a test) went on to be tested. A few (52, 8.2%) of those who said they were not asked about testing thought they were tested. Among the 52 people apparently tested without consent, 20 reported (in response to a later question) that they had had an HIV test though VCT in a government facility, so it seems they requested testing themselves, rather then the other way around. Among those who reported being tested for HIV at a government health facility, nearly all said they were given the test result.

**Table 5:** HIV testing in visits to government health facilities since August 2004

	**Weighted % respondents**	**Unweighted % respondents**	**Fraction of respondents**
Asked about being tested for HIV (offered test)	50.1	50.7	624/1232
Reported being tested for HIV:			
• Among all who visited facility	45.9	46.6	574/1232
• Among those asked about testing	83.5	83.7	522/624
• Among those not asked about testing	8.2	8.6	52/608
Given result (among those who reported being tested)	91.9	91.8	523/570

The denominator numbers indicate the number of respondents who gave information for each variable, out of the relevant totals: 1235 reporting a visit to a government health facility since August 2004; 624 offered an HIV test; 574 tested for HIV.

Among people visiting a government health facility since August 2004, men were less likely than women to say they were asked to have an HIV test (164/387 men were asked compared with 447/827 women. OR 0.63, 95% CI 0.49–0.80) and less likely to say they were actually tested for HIV (146/387 men tested compared with 415/827 women. OR 0.60, 95% CI 0.47–0.77). Among those offered testing, men were less likely than women to go on to be tested (129/164 men tested compared with 380/447 women. OR 0.65, 95% CI 0.41–1.02). Among those aged 35–54 years, 60% reported being asked to have an HIV test, while there were lower reported rates of being asked about testing in the youngest and older age groups: 45% among those aged 18–24 years, 39% among those aged 55–64 years, and 18% among those aged 65 years and above.

Most respondents (78.6%; 1198/1527) had heard about RHT in government facilities. Nearly everyone (including those who needed an explanation about RHT) said they were in favour, or strongly in favour, of RHT (94.2%; 1429/1514). Their main reasons (in response to an open-ended question) were: it encourages people to be tested (68.1%; 949/1394); people have more choices and can access treatment faster if they get tested (16.9%; 236/1394); and it helps the community by reducing the spread of HIV (16.4%; 228/1394). The main reasons among the few not in favour of RHT were: testing should be an individual choice, people should not be pushed into it (41/52); HIV testing is not the solution (7/52); and there are confidentiality problems with this testing (2/52).

Table 6 shows knowledge about where to go for an HIV test, plans to have a test, and testing within the last 12 months. Nearly all the respondents knew where to go for an HIV test. Only 12% did not plan to go for an HIV test; 88% either said they planned to have a test or responded to the question by saying they had already been tested. Although we did not ask about HIV status, a number of the people who said they had already been tested went on to explain they did not need a further test because their previous test was positive. Over half the respondents had been tested for HIV within the last 12 months. Among these, the most common place for testing was routine testing in a government facility, followed by VCT in a testing centre or attached to a government clinic. A few reported being tested privately or as part of ante-natal care.

**Table 6:** Knowledge and plans about HIV testing and actual testing within the last 12 months

	**Weighted % respondents**	**Unweighted % respondents**	**Fraction of respondents**
Know where to go for an HIV test	94.9	94.7	1451/1532
Plan to have an HIV test:			
• Yes	58.4	58.9	902/1532
• No	12.0	11.8	180/1532
• Have already been tested	29.6	29.4	450/1532
Have had a test within the last 12 months	54.9	55.3	846/1529
Routine testing in government facility*	49.2	49.9	417/836
• VCT in testing centre or government facility*	42.1	41.6	348/836
• Private facility*	4.1	4.1	34/836
• Ante-natal testing*	4.3	4.1	34/836
• Other: work, prison*	0.3	0.4	3/836

The denominator numbers indicate the number of respondents who gave information for each variable, out of the relevant totals: 1536 respondents; 846 who have had a test within the last 12 months.

* The percentages shown for place of testing are among those who reported having a test within the last 12 months and gave information about place of testing

We examined associations with several possible determinants of having an HIV test within the last 12 months. The final multivariate model is shown in Table 7. Taking other variables into account, the strongest association was with having visited a government facility within the last 12 months. A further strong association was with the belief that ART can help people with AIDS (see below). People who said they were in favour of RHT in government clinics were more than twice as likely to have been tested for HIV within the last 12 months. People under 40 years of age were more likely to have been tested, as were people who considered themselves at risk of HIV. Those without any school education were less likely to have been tested. People in rural locations were more likely to have been tested in the last 12 months than people in urban locations or in the capital.

**Table 7:** Logistic regression analysis of variables related to having an HIV test within the last 12 months

Variables	Crude OR	Adjusted OR	95% CI of adjusted OR	Mantel-Haenszel X^2^
Rural location	1.22	1.32	1.05–1.66	5.52
Male	0.49	0.55	0.43–0.69	26.45
Less than 40 years old	1.67	1.71	1.29–2.25	14.28
No school	0.63	0.68	0.47–0.97	4.48
Believe at risk of HIV	1.39	1.31	1.04–1.65	5.32
Visited government facility within last 12 m	2.73	2.47	1.86–3.27	39.80
Believe ARVs can help AIDS	2.66	1.85	1.07–3.20	4.90
In favour of routine HIV testing in govt clinics	2.65	2.01	1.14–3.51	5.92

The variables included in the original saturated model were: rural/urban location, sex of respondent, age group, education level, whether thought people with HIV should live apart, whether believed themselves at risk of HIV, whether visited a government clinic, whether believed ART could help AIDS, and whether in favour of routine HIV testing

### Knowledge and views about ART

Table 8 summarises knowledge and perceptions about ART. Almost all the respondents had heard of ART and the great majority believed ART could help people with AIDS and would recommend someone with AIDS to take ART. There was no difference between urban and rural respondents in their knowledge and views about ART. In one community where people had to travel for a day just to reach their nearest ART centre, a woman in her late 60 s explained to the team about her granddaughter's CD4 count and ART. More than half the respondents said they talked about ART in their family. Although we did not ask respondents why they believed ART could help someone with AIDS, some offered the information that they had seen people – family members, friends or neighbours – get dramatically better when taking this treatment.

**Table 8:** Knowledge and perceptions about ARVs

	**Weighted % respondents**	**Unweighted % respondents**	**Fraction of respondents**
Have heard of ART for HIV/AIDS	93.7	93.6	1427/1525
Believe ART can help someone with AIDS			
• People in their community	86.3	86.5	1321/1528
• Themselves	93.8	94.2	1441/1530
Would advise someone with AIDS to take ART	91.0	90.8	1391/1532
Talk about ART with the family			
• Often	31.3	31.1	475/1529
• Seldom	27.8	27.6	422/1529
• Never	41.0	41.3	632/1529

The denominator numbers indicate the number of respondents who gave information for each variable out of the total 1536 respondents

Focus groups in rural communities explained that they needed an entire day or even two days to collect ART from the nearest treatment centre, and said that people faced problems paying for their transport, food and even accommodation. Women said they might be stranded in the dark when trying to return home.

In urban centres, the main problem was the long queues waiting to see the doctor and then waiting to collect the medication. Even if the hospital was nearby it could still take most of the day to collect the ART. Some focus groups of youth mentioned that people did not want to be seen queuing for ART, as this would identify them as HIV positive.

Most focus groups knew about and described side-effects of ART like headaches, rashes, nightmares, vomiting and diarrhoea but said these settled down as the body got used to the treatment. Many groups referred to the belief that ART increases libido and makes people more sexually active. As one female focus group participant said: *"People on ARVs have many love affairs even if they never did that before."*

Particularly in rural communities, focus groups mentioned that people need to eat regularly when taking ART and this is difficult for very poor people who do not have enough food. Another commonly voiced concern, especially in focus groups of youth, was about people drinking alcohol while taking ART. People explained that alcohol might interact with the drugs, but more importantly they said that drinking made people forget to take their medication at the proper times. A youth focus group participant explained: *"Some take them [ARVs] to the drinking place, show them off, and still forget to take them when drunk."*

One focus group of youth in an urban community said some people sold their ART to other people in order to get money for alcohol: *"These people exchange their ARVs for money to buy alcohol or drugs." *The group alleged that the people who bought these "black market" ARVs either knew their HIV status but did not want to be seen collecting ARVs from a government facility, or were self-diagnosed but never actually tested for HIV.

The focus group discussions confirmed the strong belief in the efficacy of ART expressed by the respondents to the household interview. In the words of a male focus group participant: *"I know that ARVs do help because I have seen many sick people recover."*

When asked about other treatments people used for HIV and AIDS, many groups said that people only used ARVs. Individual participants mentioned herbal medications, other treatments from traditional practitioners, and various treatments from churches and faith healers, including cleansing by cutting the skin or purging.

## Discussion

The proportions of employed men and women in our sample were lower than in the general population according to the 2001 census, so the findings will tend to under-represent the views of employed people, especially men. However, there were no differences between employed and non-employed people in their perceptions of their HIV risk, their views about RHT, or the proportion who had been tested for HIV in the last 12 months. So the lower proportion of employed people in our sample is unlikely to have introduced an important bias in the findings. Some 29% of the households approached could not be included in the survey because no one was present and in a further 9% no one over 18 years old was present to be interviewed. Although this means we were unable to include over a third of the initially approached households, we have no particular reason to believe these "absent" households would have responded differently to the survey questions; in most cases the houses were empty because their occupants were away at their "lands".

RHT is regarded as cost-effective in resource-rich settings, even when the HIV prevalence is relatively low [16,17]. Revised guidelines from the Centers for Disease Control in the USA now recommend a routine offer of HIV testing in the majority of health care settings [18]. Some authors have stressed the need to increase the rate of HIV testing in Africa as a means of dealing with the AIDS epidemic from a public health standpoint [19,20]. Others have raised concerns that RHT might be coercive and lead to testing without consent [5], might lead to people avoiding using health care facilities because of fear of testing [6], and might lead to increased partner violence against women [21,22]. Our study suggests that RHT has been largely successful in Botswana, achieving a high rate of HIV testing without alienating users of government health facilities, and advocating for ART.

Over one half (55%) the respondents in our survey reported having an HIV test in the last 12 months. And of these, half said they had the test under the scheme of routine testing when they visited a government health facility. This is a big increase since 2005, when only 15% of those tested for HIV had been tested under the routine testing scheme [6]. HIV testing is indeed being offered to people visiting government health facilities: one half of those who attended in the last 24 months were offered testing on their last visit. The reported rate of being asked to have an HIV test was 60% among people aged 25–44 years. Perhaps of concern, the offer rate was only 45% for users aged 18–24 years, but we do not know how many of them had already been tested recently.

The RHT scheme seems to reach women more than men. Women used government health facilities more than men and, on top of this, female service users were more likely than male service users to be offered testing and to go on to be tested. This higher rate of testing under the routine testing scheme is one reason women were more likely than men to have been tested for HIV in the last 12 months. It is not clear why women visiting government clinics are more likely to be offered testing under the RHT scheme but their higher rate of acceptance of the offer is in line with previous studies about VCT. Women's take-up of VCT is generally higher than that of men and studies of different groups have reported higher HIV testing rates among women, in countries without RHT on offer [23-25]. One study in a township in Cape Town did not find a higher rate of HIV testing among women [26]. In Botswana, routinely collected data show that more women come forward for VCT than men [27] and a study of students found females were more willing than males to be tested for HIV [28].

In our study, rural dwellers were both more likely to think themselves at risk of HIV and more likely to report having an HIV test in the last 12 months. This finding may be a consequence of the widespread availability of HIV testing in Botswana: in addition to the routine testing offered in government clinics, VCT is widely available in rural areas, often attached to the government clinic in rural communities. The relative availability of ART within reach of even rural communities is probably also a factor increasing the rate of HIV testing. A recent study from Tutume in Botswana reviewed records and reported a big increase in numbers coming forward for HIV testing once ART became available locally [29]. We found an association between a positive view about ART and being tested for HIV. The association between being in favour of RHT and being tested for HIV is interesting; it could mean that people who favour RHT deliberately visit clinics so that they will be offered an HIV test.

Few people expressed concerns about the routine HIV testing policy introduced into government clinics. Indeed, nearly all respondents knew of the policy and were in favour of it. Those few people who needed an explanation of RHT were given a description of a "routine offer" approach. Some of the majority of respondents who already knew about RHT may even have believed that an "opt-out" approach was operating but nearly all of them nevertheless approved routine testing. Nearly all respondents (90%) were also comfortable about the confidentiality of the information about them in government facilities. For the 10% who were not comfortable that the information about them was kept private and confidential we have no evidence of an actual breach of confidentiality, or that their concerns were about HIV or AIDS information. Nevertheless any breach of confidentiality even in rare cases would be a serious matter given the continuing stigma around HIV and AIDS. Our findings do not suggest that people are avoiding using government facilities for fear they might be coerced into being tested for HIV: the use of government facilities is high (much higher than the use of private services) and shows no signs of reducing; and nearly all respondents said they would go to a government facility if they had an illness they thought could be due to AIDS, many specifically saying this was *because *they would be tested for HIV in the facility and could have access to ART. The association between visiting a government health facility in the last 12 months and being tested for HIV in this period could be partly because some people visited the government facility because they thought themselves at risk of HIV and wanted to be tested. The high level of reported satisfaction with the service and perception of being treated with respect in government health facilities contrast with our findings from household surveys in other countries in southern and east Africa asking similar questions [10,30,31].

There is a potential risk that instead of being a "routine offer" of testing, the routine testing becomes an opt-out process, whereby one is tested unless specifically requesting not to be so. This seems to be only rarely the case in Botswana. Very few people thought they were tested despite not being asked. Some people (20 of the 52 tested "without consent") apparently went to the facility specifically to *request *testing (essentially using the clinic for VCT) and reported that they were not *asked *for testing. Others were older individuals who thought they would have been tested anyway and reported being given a test result, when they simply had blood taken and were given the results of a different type of test. There could be potential for false reassurance in such cases, and it is important that people do not believe they have tested negative for HIV when they have not in fact been tested. Most (83.5%) of those who were offered HIV testing said they had the test; women in particular rarely refused the testing offer. This high rate of acceptance of the HIV testing offer could reflect a tendency to do what health care workers instruct, which has been raised as a concern about the routine testing system [5,21]. It is also possible that some people who were asked about having an HIV test declined to have the test but were nevertheless tested against their will. However, we had no indication from any respondent that this happened and we believe it is unlikely.

Nearly everyone who reported being tested for HIV when they visited a government clinic also reported being given the result. The few who did not get a result could have been waiting to receive it after the blood was sent away for testing. The usual practice for HIV testing in government health facilities is to use a rapid testing method and give the result immediately. However, if the facility runs out of rapid testing kits, they send the blood to be tested elsewhere. At least one young woman who reported being tested but not getting the result admitted to the interviewer that she "ran away" because she found she could not face getting the result.

We found that both men and women were more likely to think themselves at risk of HIV if they had suffered violence from their partner in arguments in the last year. This is in line with studies that have reported a higher rate of HIV infection among women who have suffered gender-related violence [32]. It has been reported that women known or suspected to be HIV positive can suffer violence and abuse as a result [33] and fear of violent reactions from partners can be a barrier to women being tested for HIV [22]. We did not find any association between suffering partner violence and having had an HIV test in the last 12 months. In this cross-sectional study we would not have been able to say which came first, the testing or the violence. We cannot rule out that women who experienced violence are less likely to visit clinics (and be tested) but at the same time women who are tested are more likely to experience partner violence afterwards.

According to government figures, in February 2006 some 61,981 people in Botswana were receiving ART, with 51,203 of them receiving treatment through the public sector and 2,460 out-sourced from the public sector [34]. According to a WHO report, by the end of 2005, some 85% of people in Botswana in need of treatment were receiving ART [35]. By comparison, in South Africa in 2005, less than 20% of the almost one million people in need of ART were receiving it [1]. Our study suggests that the Botswana public is now fully convinced of the value of ART. This seems to be in large part because the widespread treatment provision means many people have seen relatives or friends "come back from the dead" while taking ART. Word of mouth testimony spreads quickly in Botswana, where there are strong family and community ties within the small population.

Corruption in health services is a well-known problem in many developing countries. In this study we did not ask about unofficial payments to health workers as part of the individual questionnaires, but payments to health workers or health workers stealing the medicines were not raised at all as concerns when the focus groups discussed problems of access to ART. Only one youth focus group mentioned that some people sold their ARVs in order to get money for alcohol or drugs.

The ART programme in Botswana is not confined to urban or peri-urban areas. Our study found knowledge and approval of ART to be high even in remote communities where people have to travel long distances to get their ART. The main complaint about ART was the long travel some people had to make to get their treatment, and this is especially a problem for people in remote rural communities.

One concern about ART and a very high prevalence of HIV infection is that people taking ART remain sexually active and may continue to spread the virus [36], whereas people sick with AIDS may be too ill to be sexually active. There is a common belief, voiced in the focus groups in this study, that ART actually stimulates sexual drive. However, other authors have reported a reduction in risky sexual behaviour in a programme of ART combined with counselling in Uganda [37].

Despite these successes in encouraging HIV testing and advocating and implementing ART, the HIV epidemic in Botswana remains a crucial public health challenge: unless the HIV incidence falls significantly the number of people living with HIV will actually increase as ART prolongs the life of those already infected. While VCT and RHT are clearly important for tertiary prevention, the role of VCT for secondary prevention is less clear, since behaviour change among people coming forward for VCT is mostly limited to those who test positive [38,39]. The government of Botswana now needs to build on its successful tertiary prevention programme, which has given it considerable credibility with the public, to face the challenges of secondary, and particularly primary, prevention.

## Conclusion

In 2006, public awareness about and approval of RHT in government health facilities was very high. The high rate of RHT has contributed to the overall high rate of HIV testing. At the same time, there is a very high public awareness about ART throughout the country and a near universal perception that ART can help someone with AIDS. The Botswana government's programme to increase HIV testing and uptake of ART is apparently working well. However, on its own, it may not be enough to turn the tide of the epidemic. This will require further concerted efforts to reduce the rate of new HIV infections.

## Abbreviations

AIDS Acquired immune deficiency syndrome

ART Antiretroviral therapy

ARVs Antiretrovirals

CIET Community Information Empowerment and Transparency

HIV Human immunodeficiency virus

RHT Routine HIV testing

VCT Voluntary counselling and testing (for HIV)

## Competing interests

The author(s) declare that they have no competing interests.

## Authors' contributions

AC collaborated in the study design, organised the fieldwork, performed the statistical analysis and drafted the manuscript. NA conceived of the study, led the design, advised on the analysis and helped to draft the manuscript. DM helped to organise the fieldwork, organised the data management, assisted with analysis, and helped to draft the manuscript. TM helped to organise the fieldwork, trained fieldworkers, and helped with data management. MM participated in design, helped to train fieldworkers, helped to organise the fieldwork, and participated in analysis discussions. All authors read and approved the final manuscript.

## Pre-publication history

The pre-publication history for this paper can be accessed here:


